# Nobiletin with AIEE Characteristics for Targeting Mitochondria and Real-Time Dynamic Tracking in Zebrafish

**DOI:** 10.3390/molecules28124592

**Published:** 2023-06-06

**Authors:** Tingting Jin, Na Li, Yi Wu, Ying He, Depo Yang, Feng He

**Affiliations:** School of Pharmaceutical Science, Sun Yat-sen University, Guangzhou 510006, China; jintt8@mail2.sysu.edu.cn (T.J.); lina49@mail2.sysu.edu.cn (N.L.); wuyi76@mail2.sysu.edu.cn (Y.W.); heying67@mail2.sysu.edu.cn (Y.H.); lssydp@mail.sysu.edu.cn (D.Y.)

**Keywords:** nobiletin, AIEE, zebrafish, visual pharmacokinetics

## Abstract

Nobiletin is a natural product with multiple physiological activities and is the main ingredient of *Pericarpium Citri Reticulatae*. We successfully discovered that nobiletin exhibits aggregation induced emission enhancement (AIEE) properties and it has significant advantages such as a large Stokes shift, good stability and excellent biocompatibility. The increase in methoxy groups endows nobiletin a greater fat-solubility, bioavailability and transport rate than the corresponding unmethoxylated flavones. Ulteriorly, cells and zebrafish were used to explore the application of nobiletin in biological imaging. It emits fluorescence in cells and is specifically targeted at mitochondria. Moreover, it has a noteworthy affinity for the digestive system and liver of zebrafish. Due to the unique AIEE phenomenon and stable optical properties of nobiletin, it paves the way for discovering, modifying and synthesizing more molecules with AIEE characteristics. Furthermore, it has a great prospect with regard to imaging cells and cellular substructures, such as mitochondria, which play crucial roles in cell metabolism and death. Indeed, three-dimensional real-time imaging in zebrafish provides a dynamic and visual tool for studying the absorption, distribution, metabolism and excretion of drugs. In this article, more directions and inspiration can be presented for the exploration of non-invasive pharmacokinetic research and intuitive drug pathways or mechanisms.

## 1. Introduction

As a highly sensitive and noninvasive technique, fluorescence imaging has been widely used in biomedicine, which can monitor complex physiological activities, such as pathological changes, biological processes, drug monitoring and so on [1]. However, traditional organic fluorescent materials are quenched at high concentrations or in solid states. The notorious photophysical phenomenon, known as aggregation-caused quenching (ACQ) [2], greatly limits the practical application of luminescent materials. Tang’s group first discovered the phenomenon called aggregation-induced emission enhancement (AIEE) in 2001, which is completely opposite to the ACQ effect [3]. Compounds with AIEE characteristics exhibit no fluorescence or extremely weak fluorescence in dilute solutions, while the fluorescence has a significant enhancement in aggregated or solid states. Compared with traditional organic fluorescent dyes, AIEE molecules have numerous significant advantages, such as a high emission efficiency, large Stokes shift, excellent photostability and biocompatibility, low background noise and impressive photobleaching resistance and preeminent signal reliability [4,5]. These AIEE molecules are ideal candidates for subsequent fluorescence tracking after drug administration in target cells and live animals [6].

**Figure 1:**
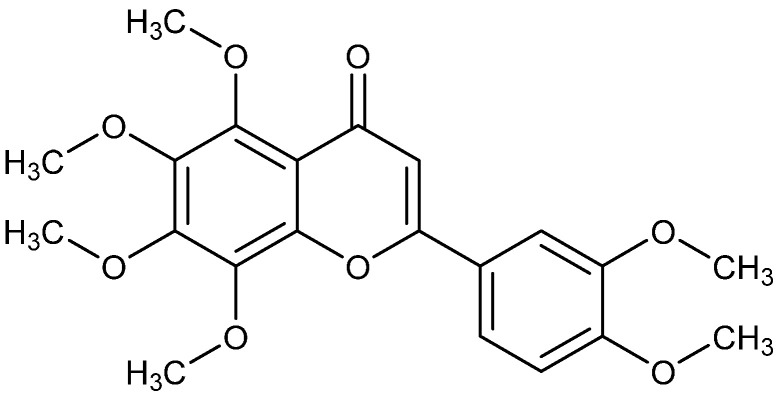
The structure of nobiletin.

*Pericarpium Citri Reticulatae* is one of the most famous Chinese medicinal herbs and is extensively used due to its excellent pharmacological effects, unique aroma and low toxicity [7]. As a natural plant-derived product, nobiletin has received widespread attention due to its excellent pharmacological activities, such as anti-tumor, anti-inflammatory, antioxidant and neuroprotective properties [8,9]. Based on our previous specialized work on the AIEE properties of flavonoids, most of the flavonoids with AIEE characteristics that we have identified contain the methoxy group [10,11]. Fortunately, we have discovered nobiletin, a representative of polymethoxy flavonoid compounds, which exhibits significant AIEE characteristics. Meanwhile, nobiletin, one of the methoxylated flavonoids, exhibits much higher metabolic stability, lipid solubility and transport rates than corresponding unmethylated flavonoids [12] on account of its increase in liposoluble polymethoxy groups, which has also prompted us to explore its properties in cells and zebrafish. The structure of nobiletin is shown in Figure 1.

In recent years, zebrafish, as highly promising clinical models along with vertebrates, have received increasing attention. Its main features include its high homology with human genes, low cost and easy reproduction, abundant and available transgenic lines, effective combination with high-throughput screening technology, rapid growth and development, complete establishment of main organs within a week and transparent body for easy imaging for transparent [13,14]. As a stable and accessible evaluation model, zebrafish models have the advantages of simplicity, efficiency and visualization, which can commendably bridge the gap between in vitro cell models and in vivo mammalian models [15,16]. The zebrafish imaging can achieve nearly real-time pharmacokinetic research, allowing researchers to directly observe the dynamic changes in fluorescent drugs in vivo [17]. In addition, it can be stanchly combined with high-throughput screening technology to evaluate the absorption, distribution, metabolism and excretion of drugs under different conditions [18], which provides a real-time and visual platform for pharmacokinetic research.

Based on previous research, for nobiletin, the liver and digestive tract are the main metabolic sites [19]. The protective function of the liver may be related to its antioxidant, anti-inflammatory, immunomodulatory and anti-fibrotic activities [20]. Other studies have shown that flavonoid supplements mainly prevent dyslipidemia of the liver by inhibiting fatty acid synthesis and increasing fatty acid oxidation, regulating the detoxification pathway and inhibiting oxidative stress in liver cells [21,22]. The gastrointestinal tract is the largest interface of the body and plays a crucial role in regulating immune homeostasis [23]. Among the beneficial effects of the gastrointestinal tract, flavonoids have been identified to maintain the integrity of the intestinal barrier, regulate the gastrointestinal immune system, increase the proportion of beneficial bacteria and so on [24]. In our experiment of zebrafish real-time tracking, the targeted distribution and metabolism of nobiletin in the gastrointestinal tract were captured, paving the way for further research on the pathways and mechanisms of nobiletin.

## 2. Results and Discussion

### 2.1. Photophysical Properties

**Figure 2:**
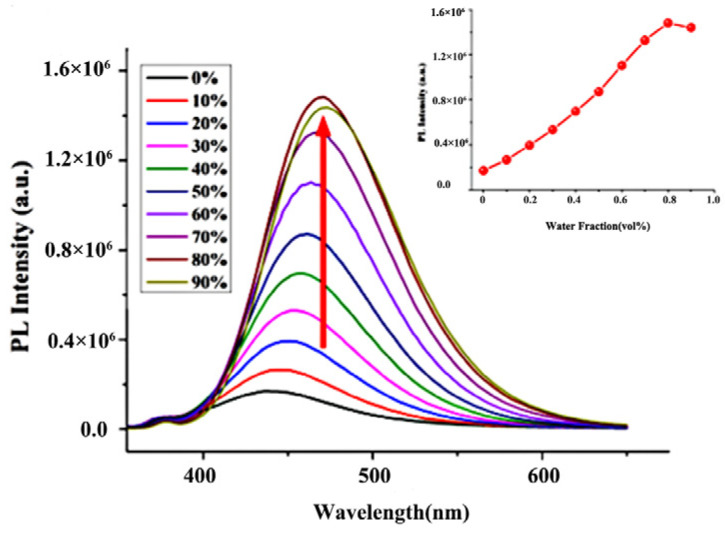
Fluorescence spectra and variation of maximum fluorescence intensity of nobiletin at different *f_w_* values in CH_3_OH/H_2_O (c = 20 μM).

**Figure 3:**
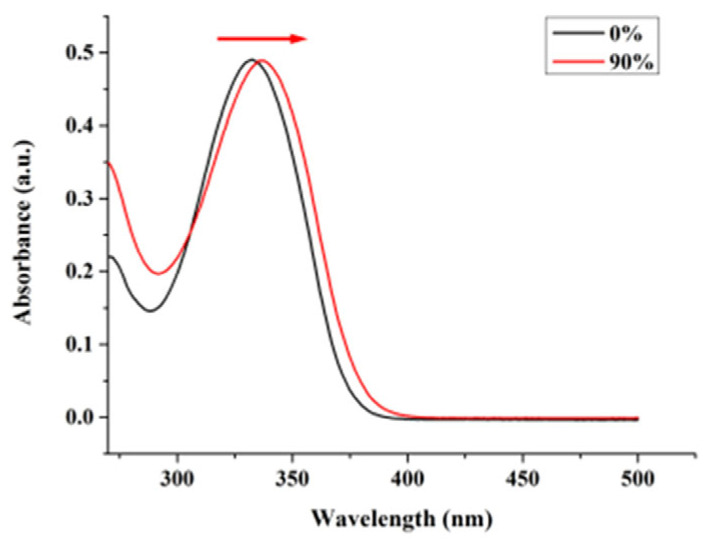
Absorption spectra of nobiletin in pure CH_3_OH and in CH_3_OH/H_2_O mixed solution of *f_w_* 90%.

**Figure 4:**
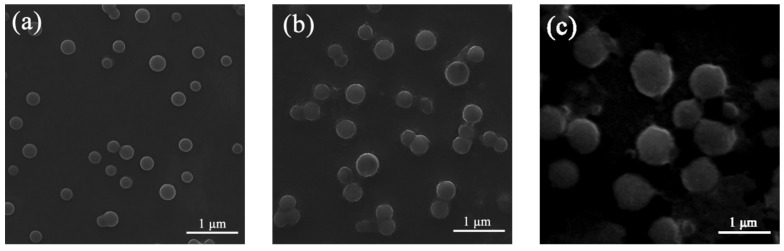
(**a**) SEM image of nobiletin in CH_3_OH/H_2_O (*v*:*v* 5:5). (**b**) SEM image of nobiletin in CH_3_OH/H_2_O (*v*:*v* 2:8). (**c**) SEM image of nobiletin in CH_3_OH/H_2_O (*v*:*v* 1:9).

**Figure 5:**
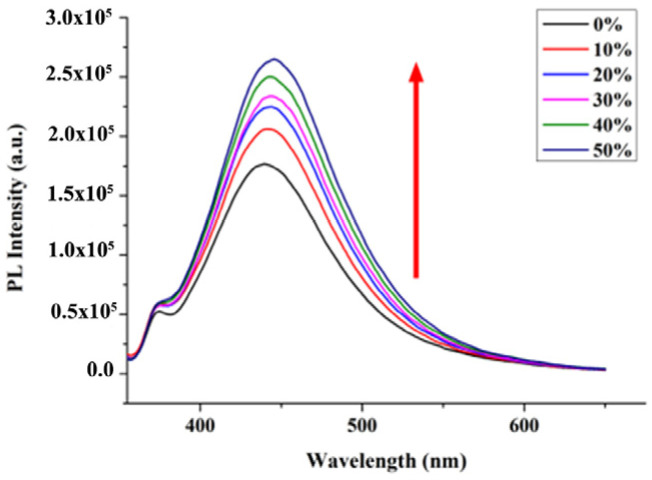
Fluorescence spectra of nobiletin in CH_3_OH/EG mixed solutions (*v*:*v*).

The optical properties of nobiletin were determined by UV–vis absorption and a fluorescence spectrometer. The results are summarized in Figure 2, Figure 3, Figure 4 and Figure 5. The nobiletin is soluble in most organic solvents but insoluble in water. It can be observed from the results that in the methanol/water mixed solution, when the fraction of water (*f_w_*) was 0%, it exhibited weak fluorescence. Furthermore, when *f_w_* further increased, the nobiletin gradually formed nanoaggregates in the poor solvents and the fluorescence intensity gradually increased. As *f_w_* changed from 0% to 80%, the fluorescence intensity of nobiletin increased by about 10 times and the red-shift shifted from 437 nm to 471 nm. For nobiletin, after the fluorescence intensity reached the maximum values at 80%, respectively, the fluorescence intensity decreased slightly, which may have been due to the changes in the packing modes of molecule.

The absorption spectra of nobiletin were observed in pure methanol and in methanol/water mixed solutions of *f_w_* 90% at room temperature. As can be seen in Figure 3, the absorption peak of nobiletin was 332 nm, respectively, in the pure methanol solution. When *f_w_* changed to 90%, there was a significant red shift in the absorption spectra and the absorption peak changed to 337 nm, respectively.

The Scanning Electron Microscopic (SEM) study was collected in Figure 4. When *f_w_* was 50%, the particles in the solution were spherical particles with a uniform distribution and small diameter. This is due to the aggregation of nobiletin to form particles when the poor solvents were added. When *f_w_* increased to 80%, the spherical particles in the solutions became lager and still uniformly distributed in the solution. Compared with the fluorescence spectra, when *f_w_* gradually increased, the fluorescence became stronger step by step and the spherical particles in the solutions became larger. For nobiletin, which can be seen in Figure 4c, the irregular particles appeared in the solution when *f_w_* was 90%, which may be a reason for the decrease in fluorescence. The changing trend of the SEM result is consistent with that of fluorescence spectra. Combining the maximum absorption shift and the results of SEM indicates that fluorescence enhancement is related to the formation of aggregates.

To gain a deeper understanding of AIEE characteristics of nobiletin, the fluorescence quantum yield was measured both in pure methanol solution and in the methanol/water mixture. As can be seen in Table 1, the fluorescence quantum yield of nobiletin was low in the pure methanol solution. When *f_w_* increased, the fluorescence quantum yield rose simultaneously. The changing trend of the fluorescence quantum yield is also consistent with the fluorescence spectra.

In past years, many researchers have worked on the AIEE mechanisms. Based on our previous research on AIEE properties of other flavonoids, we used a viscosity test to study the AIEE mechanism of nobiletin. As shown in Figure 5, the fluorescence gradually increased as the content of ethylene glycol in the mixture changed from 0 to 50%. The increase in solution viscosity limits the intramolecular rotation (RIR) of the compound [25,26], which leads to the enhancement of fluorescence, indicating that restriction of RIR may be the main reason for the AIEE characteristic of nobiletin.

**Figure 6:**
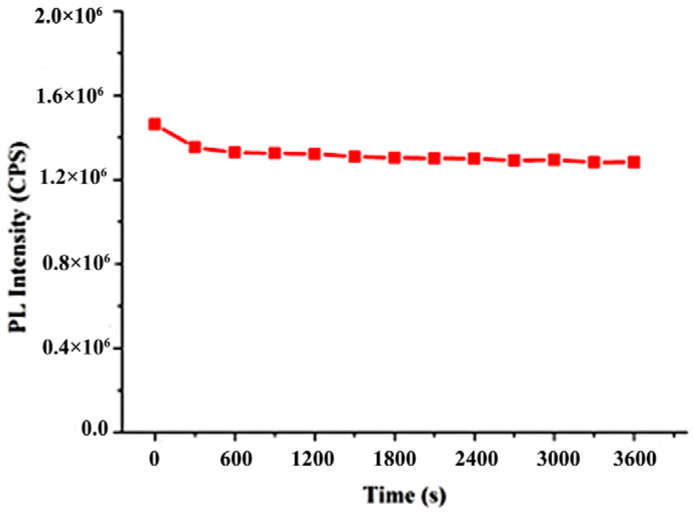
The changes in fluorescence intensity of nobiletin over time in CH_3_OH/H_2_O (*v*:*v* 1:9) mixed solutions.

Fluorescence stability is an important indicator for evaluating the fluorescence performance of compounds and it is also a vital reference standard for whether a compound can be applied practically. Therefore, we measured the fluorescence intensity of the mixed solutions of nobiletin. As can be seen in Figure 6, the fluorescence intensity of nobiletin was only slightly decreased in the first 10 min of the experiment and then maintained stable in the next 50 min. Thus, the nobiletin shows outstanding fluorescence stability.

### 2.2. Cell Imaging

**Figure 7:**
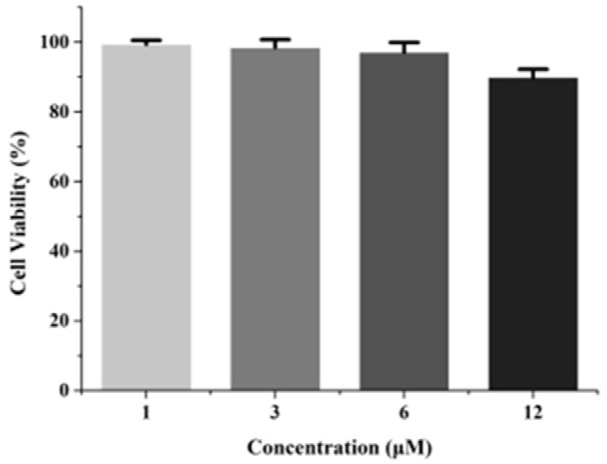
Cell viability of PC12 cells treated with different concentrations of nobiletin for 24 h.

To evaluate the biomedical application potential of nobiletin, the cell viability of nobiletin toward PC12 cells were examined by an MTT assay. Figure 7 showed the viability values of PC12 after they were treated with different concentrations of nobiletin for 24 h. It can be seen in Figure 7 that the cell viability was still more than 80% with a 24 h incubation time at each concentration, indicating that it shows a low cytotoxicity to PC12. Low cytotoxicity and good biocompatibility confer the nobiletin with an immense potential for biochemical application.

**Figure 8:**
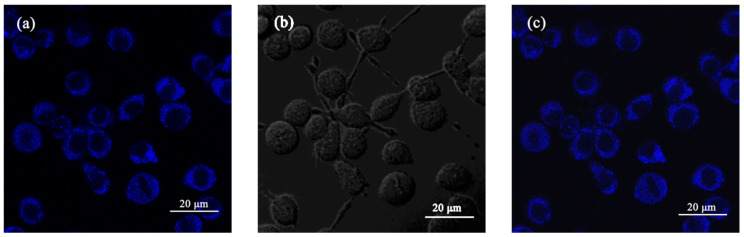
(**a**) The fluorescence image of nobiletin in PC12 cells. (**b**) The bright field image. (**c**) The merge image of (**a**,**b**). The excitation wavelength was 359 nm. The collected wavelength range was 430–470 nm.

The cell uptake behavior of nobiletin was further examined by Confocal Laser Scanning Microscope (CLSM) to evaluate the biological imaging potential of nobiletin. As shown in Figure 8, after incubating with 20 μM nobiletin for 30 min, the cell morphology had no significant change and strong blue fluorescence was observed in the cytoplasm. These results suggested that nobiletin possesses good biocompatibility and can penetrate cell membranes, indicating that it can potentially be used in cell bioimaging.

**Figure 9:**
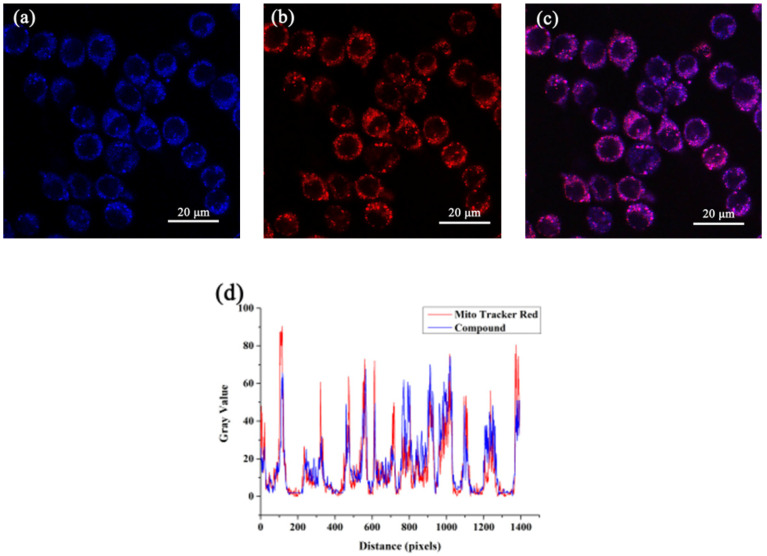
Co-localized images of PC12 cells incubated with (**a**) (20 μM) and Mito Tracker Red (200 nM) (**b**) for 40 min. The merged image stained with (**c**). The Pearson correlation coefficients of nobiletin with (**d**). The excitation wavelength of nobiletin was 359 nm and the collected wavelength was 471 nm. The excitation wavelength of Mito Tracker Red was 578 nm and the collected wavelength range was 570–620 nm.

In past years, some research groups have reported that flavonoids show a specific targeting effect on mitochondria and can regulate their functions [27,28,29]. So, in order to verify whether nobiletin accumulate in mitochondria, PC12 cells were treated with 20 μM nobiletin and 200 nM Mito Tracker Red for 40 min. As shown in Figure 9, nobiletin showed strong blue fluorescence and Mito Tracker Red showed strong red fluorescence. The areas it stained were highly coincident. In order to accurately analyze the coincidence degree of the two, image J was used for quantitative analysis. Furthermore, the Pearson correlation coefficients of nobiletin were calculated to be 0.85, respectively. The results showed that the staining regions of it were highly coincident with that of Mito Tracker Red, indicating that nobiletin could specifically accumulate at the mitochondria.

### 2.3. Visible and Dynamic Distribution of Nobiletin in Zebrafish

The genome of zebrafish is highly homologous to that of humans; they can be handled easily given their small size and their embryos and larvae are transparent [30,31,32]. Due to the strong blue fluorescence of nobiletin in cell imaging, we chose to use zebrafish to explore their potential application in animal imaging. The zebrafish embryos were immersed in the nobiletin solution (80 μM) for 0.5 h and in the culture solution for 1.5 or 3 h and were then observed, which indicated that the nobiletin can penetrate the zebrafish embryo membrane and enter the body.

**Figure 10:**
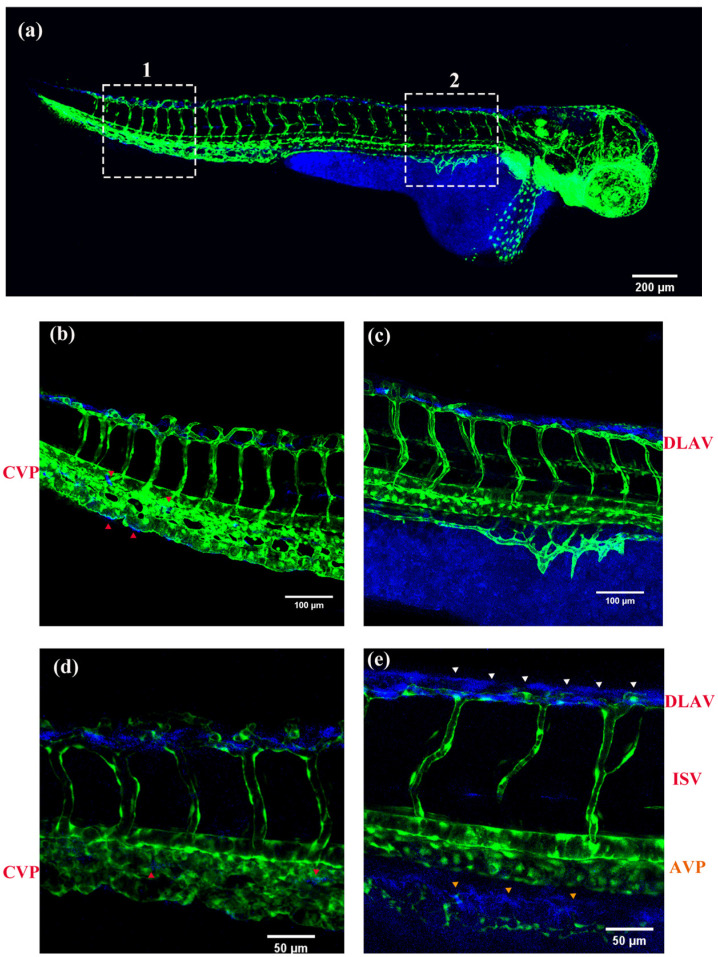

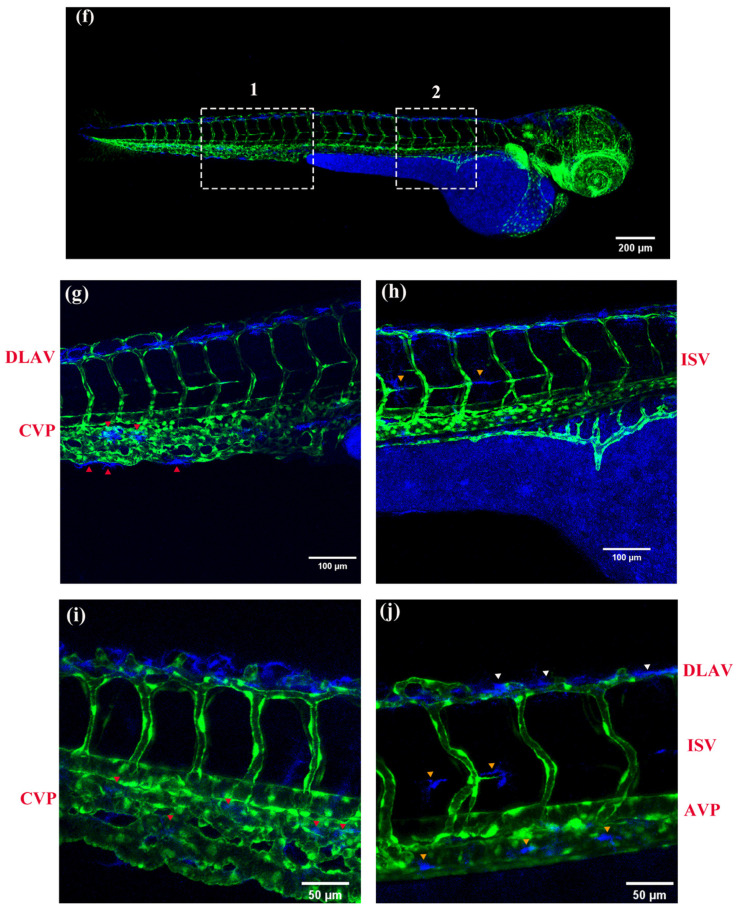
Fluorescence images of zebrafish embryos at 48 hpf after soaking in the mixed solutions (80 μM) of nobiletin. The excitation wavelength was 359 nm and the collected wavelength was 471 nm. (**a**) Refers to 48 hpf zebrafish soaked in the nobiletin solution for 0.5 h and in culture solution for 1.5 h. (**b**) A 20× magnification of the dashed box 1 in (**a**). (**c**) A 20× magnification of the dashed box 2 in (**a**). (**d**) A 40× magnification of the dashed box 1 in (**a**). (**e**) A 20× magnification of the dashed box 2 in (**a**). (**f**) Refers to 48 hpf zebrafish soaked in the nobiletin solution for 0.5 h and in culture solution for 3 h. (**g**) A 20× magnification of the dashed box 1 in (**f**). (**h**) A 20× magnification of the dashed box 2 in (**f**). (**i**) A 40× magnification of the dashed box 1 in (**f**). (**j**) A 40× magnification of the dashed box 2 in (**f**). (DLAV: dorsal longitudinal anastomosis of vessels; ISV: intersegmental vessels; PCV: posterior caudal vessels; AVP: anterior venous plexus; CVP: caudal vein plexus).

As shown in Figure 10, it can be seen that the blue fluorescence is mainly distributed in the yolk sac and in dorsal longitudinal anastomotic blood vessels of the zebrafish embryos at 48 hpf (hours post fertilization). There is also a small amount of distribution in the tail vein capillaries, which indicated that the nobiletin can penetrate the zebrafish embryo membrane and enter the body. Through the comparison of (b) and (g) as well as between (d) and (i), it can be seen that the prolonged circulation time leads to a significant increase in the distribution of drugs in the caudal vein plexus (CVP), marked by a red triangle. By comparing (c) and (h) as well as (e) and (j), it can be observed that the circulation time is prolonged, that the tissues around the segmental blood vessels begin to have drug distribution and drugs also appear in the posterior main vein, while the drugs in dorsal longitudinal anastomosis of vessels (DLAV) are significantly reduced. During this period, the dynamic distribution changes in the drugs in zebrafish can be observed, providing valuable reference for further clinical research on drug absorption and distribution.

**Figure 11:**
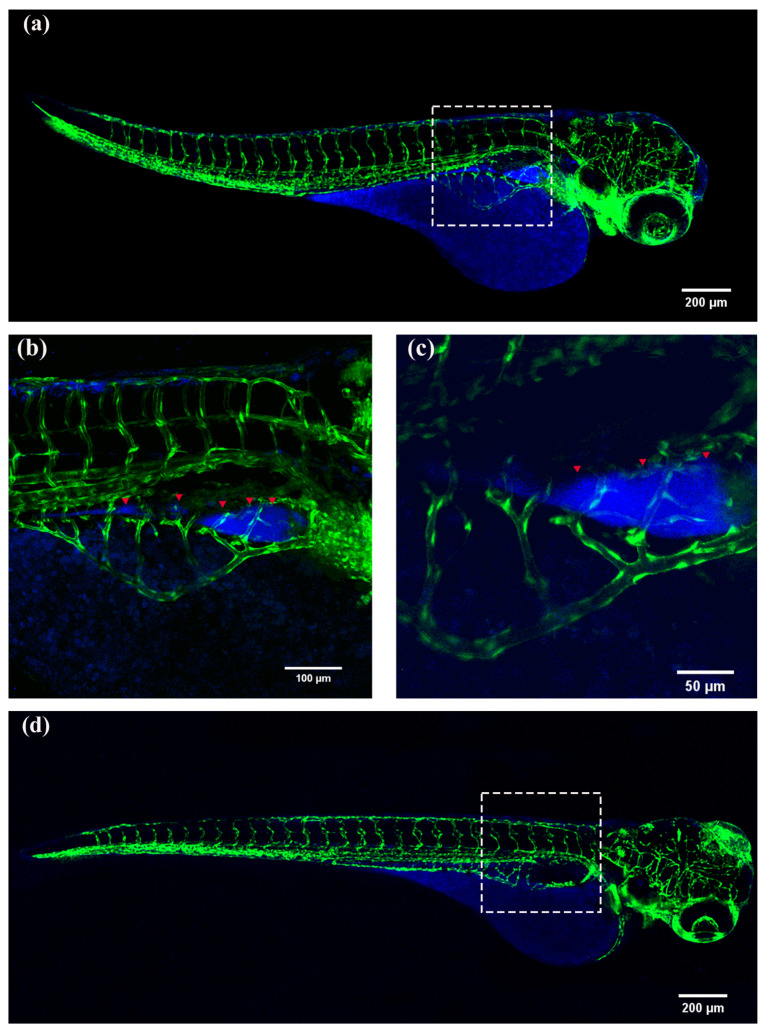

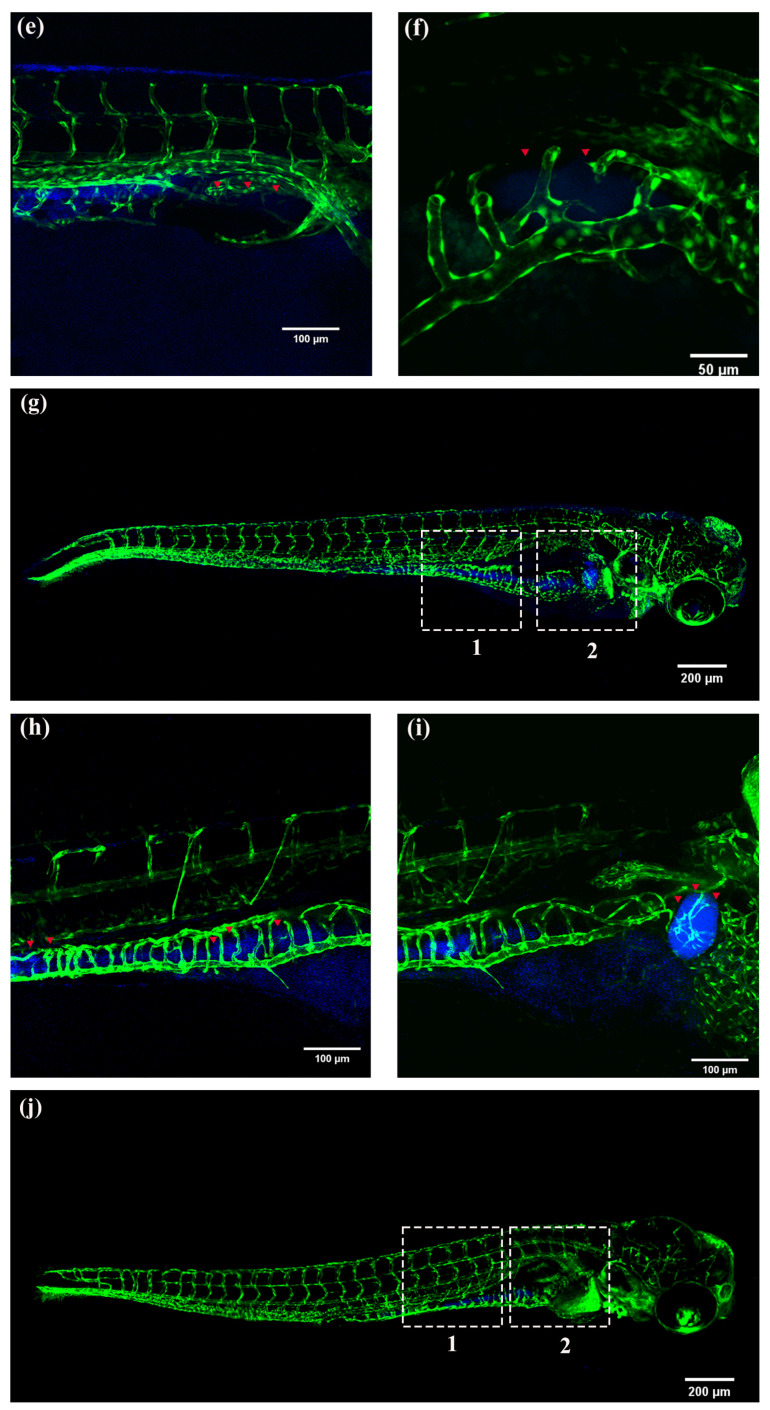

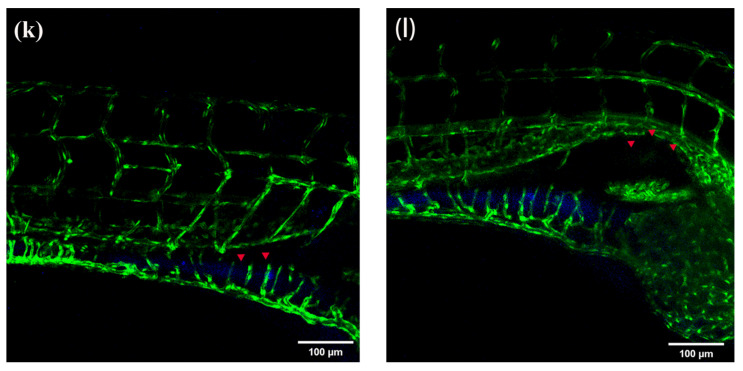
Fluorescence images of zebrafish embryos at 72 hpf and 144 hpf after soaking in the mixed solutions (80 μM) of nobiletin. The excitation wavelength was 359 nm. The collected wavelength was 471 nm. (**a**) Refers to 72 hpf zebrafish soaked in the nobiletin solution for 0.5 h and in culture solution for 1.5 h. (**b**) A 20× magnification of the dashed box in (**a**). (**c**) A 40× magnification of the dashed box in (**a**). (**d**) Refers to 72 hpf zebrafish soaked in the nobiletin solution for 0.5 h and in culture solution for 3 h. (**e**) A 20× magnification of the dashed box in (**d**). (**f**) A 40× magnification of the dashed box in (**d**). (**g**) Refers to 144 hpf zebrafish soaked in the nobiletin solution for 0.5 h and in culture solution for 1.5 h. (**h**) A 20× magnification of the dashed box 1 in (**g**). (**i**) A 20× enlarged image of white dashed box 2 in (**g**). (**j**) Refers to 144 hpf zebrafish soaked in the nobiletin solution for 0.5 h and in culture solution for 3 h. (**k**) A 20× magnification of the dashed box 1 in (**j**). (**l**) A 20× magnification of the dashed box 2 in (**j**).

With further development, various organs of zebrafish begin to mature. The yolk sac of 72 hpf zebrafish larvae still contain a large amount of fat, except for the yolk sac, where drugs are distributed in the liver, which can be observed through Figure 11. By prolonging the circulation time, it can be seen that the drug fluorescence in the liver is significantly reduced, as shown by the comparison between (b) and (e) and that between (c) and (f) in Figure 11. It indicates that the liver of zebrafish with 72 hpf has a basic development and can complete drug metabolism within a short period of time.

The fat in the yolk sac of 144 hpf zebrafish eggs is almost consumed completely and the organ system gradually develops and to be mature. The aggregation and distribution of drugs in the liver and intestines of zebrafish are shown in Figure 11g–i. As the circulation time increases, most of the drugs in the liver and intestines are metabolized, as shown in Figure 11j–l. Through the experimental results, it can be established that the organs of 144 hpf zebrafish have basically developed and matured and that the drug is specifically distributed in the liver and intestines and is rapidly metabolized in a short period of time.

## 3. Materials and Methods

### 3.1. Materials and Instruments

All the regents and solvents were of analytical grade, purchased from commercial suppliers and used without further purification. Methanol solution, dimethylsulfoxide (DMSO), nobiletin (nobiletin) and ethylene glycol were purchased from Aladdin (Shanghai, China). PC12 cells were obtained from ATCC. Rosewell Park Memorial Institute 1640 medium (RPMI-1640), fetal bovine serum (FBS), 0.25% trypsin solution, penicillin-streptomycin solution, 3-(4,5-dimethyl-2-thiazolyl)-2,5-diphenyl-2-H-tetrazolium bromide (MTT), Mito Tracker Red assay kit (MT) and phosphate buffered saline (PBS) were purchased from Thermo-Fisher Biochemical Products (Beijing, China). Fluorescence spectra and absolute fluorescence quantum yields were collected from a Fluoromax-4 spectrophotometer (HORIBA Instruments Incorporated, Kyoto, Japan). Ultraviolet (UV) absorption spectra were collected from a UV-2600 spectrometer (Shimadzu, Kyoto, Japan). Scanning electron microscope (SEM) images were obtained from a Quanta 400F thermal field emission environmental scanning electron microscope (FEI/OXFORD/HKL, Eindhoven, The Netherlands). Zebrafish images were obtained from a FV3000 confocal laser scanning microscope (OLYMPUS, Tokyo, Japan). Cell viability was analyzed by a Synergy H1 microplate reader (BioTek, Shoreline, WA, USA). Fluorescent images of zebrafish embryos were performed on the image j (Fuji, Santa Clara, CA, USA).

### 3.2. Preparation of UV–Vis Spectra and PL Spectra, SEM and Viscosity Measurements

#### 3.2.1. Preparation of UV–Vis Spectra and PL Spectra

The nobiletin was accurately weighed as 1.68 mg, respectively, and dissolved in a 20 mL volumetric flask with methanol solution to prepare a mother liquor with a concentration of 200 μM. Then, it was diluted 10 times with methanol and water to prepare mixed solutions with different water fractions (*f_w_* = 0, 10, 20, 30, 40, 50, 60, 70, 80 and 90%). The final concentration of the mixed solution was 20 μM. Subsequently, the UV–vis absorption spectra and fluorescence spectra were measured at room temperature after 10 min sonication of the mixed solution. The excitation wavelength of fluorescence spectra was 335 nm. The excitation slit and emission slit were both set at 5.0 nm. The preparation of the fluorescence quantum yield measurement and the fluorescence stability were the same as above.

#### 3.2.2. Preparation of SEM Measurements

The preparation of the mixed solution of the compound was the same as above. After sonication, the mixed solutions with different water fractions were dropped, respectively, on the silicon wafer. Then, they were placed at room temperature for 24 h to evaporate. Finally, they were measured by a scanning electron microscope.

#### 3.2.3. Preparation of Viscosity Measurements

Nobiletin was prepared to methanol solutions with a concentration of 200 μM. Then, it was diluted with ethylene glycol (EG) and methanol to prepare CH_3_OH/EG mixed solutions with different EG fractions (*f_e_* = 0, 10, 20, 30, 40 and 50%). The final concentration of the mixed solutions was 20 μM. Subsequently, the mixed solutions were sonicated for 10 min and then they were measured by a fluorescence spectrometer.

### 3.3. Cell Culture

PC12 cells were cultured in RPMI-1640 culture medium supplemented with 10% FBS, 100 μg/mL streptomycin and 100 U/mL penicillin in a cell incubator with 5% CO_2_ at 37 °C.

### 3.4. Cell Viability Evaluations

PC12 cells were seeded in 96-well microplates at a density of 5 × 10^4^ cells mL^−1^. After 24 h, the cells were incubated with 1, 3, 6 and 12 μM nobiletin for 24 h. Subsequently, 20 μL MTT was added to each well and the plates were incubated for another 4 h. Then, the solutions of each well were removed and 150 μL DMSO was added to each well. After shaking the plates for 10 min, the absorbance at 490 nm was measured by a microplate reader.

### 3.5. Cell Imaging

PC12 cells were seeded in glass bottom dishes with a density of 1 × 10^5^ per dish. After 24 h, the cells were incubated with nobiletin, respectively, at a concentration of 20 μM for 30 min at 37 °C. Subsequently, the cells were washed three times with PBS to remove the compound. Then, the cell imaging was taken with a confocal laser scanning microscope with an excitation wavelength of 359 nm. The preparation of co-localization cell imaging was similar to that above. On the day of treatment, the cells were treated with a compound at a concentration of 20 μM and with Mito Track Red at a concentration of 200 nM for 40 min at 37 °C. Then, the cells were washed three times with PBS and observed by a confocal laser scanning microscope.

### 3.6. Zebrafish Imaging

Zebrafish (Tg(fli1a: EGFP)) were obtained from the Zebrafish Resource Center (Core Lab Plat for Medical Science, Zhongshan School of Medicine, Sun Yat-sen University). All procedures for this study were approved by the Animal Ethical Experimentation Committee of Sun Yat-sen University and followed the National Institute of Health guidelines for the care and use of animals. The ethics approval number is SYSU-IACUC-2020-B0659. Zebrafish were cultured at 28.5 °C with a 10/14 h dark/light cycle. The 48 hpf zebrafish embryos were randomly divided into two groups. Subsequently, they were treated with nobiletin (80 μM), respectively, for 0.5 h, then washed three times with PBS to remove the compound and remained for 1.5 or 3 h at 28.5 °C. The imaging experiments were carried out by a confocal laser scanning microscope. The other zebrafish imaging experiments were the same as that described above.

## 4. Conclusions

In summary, we have successfully discovered the prominent AIEE characteristics of nobiletin, which exhibits a large Stokes shift and good photostability. The excellent biocompatibility was demonstrated thoroughly with the cell viability assessment. In addition, further research was conducted on the applications of nobiletin in the biomedical field in cell imaging and zebrafish tracking. Cell imaging showed that nobiletin is fat-soluble and can easily cross cell membranes. There is no significant change in the morphology of PC12 cells, which hints that they may have great application prospects in cell imaging. In the mitochondria co-localization experiment, highly overlapping areas and correlation coefficients were observed, signaling that nobiletin has a specific targeting effect on mitochondria.

In addition to the imaging application of cells in vitro, the further exploration of nobiletin in complex live animals is much more anticipated, therefore, we chose zebrafish as the model animals. Surprisingly, blue fluorescence can also be observed in the liver and gastrointestinal tract of zebrafish, which may be related to the anti-inflammatory and antioxidant effects. In zebrafish of different sizes and cycle times, changes in drug absorption, distribution, metabolism and excretion can be observed in real time, which provides direction for further real-time visualization of drug clinical pharmacokinetics research.

Mitochondria play an important role in the development and pathogenesis of non-alcoholic fatty liver disease (NAFLD), which is also associated with metabolic syndrome, diabetes and so on. In our experiments, the fluorescence of nobiletin was observed in both mitochondria and the liver of zebrafish, providing a solid foundation for further exploration of the intrinsic relationship between mitochondria and HALFD, which will contribute to exploring the mechanisms of related diseases.

## Figures and Tables

**Table 1:** The fluorescence quantum yield of nobiletin.

Compound	Solvent	Quantum Yield (φF)
	CH_3_OH	0.04
nobiletin	CH_3_OH/H_2_O (2:8)	0.12
	CH_3_OH/H_2_O (1:9)	0.11

## Data Availability

Not applicable.
